# Caregiver App Use and Child Behavioral Adherence in Relation to Obesity-Related Outcomes in an mHealth App-Assisted Childhood Obesity Intervention: Secondary Analysis of a Cluster Randomized Trial

**DOI:** 10.2196/89548

**Published:** 2026-09-24

**Authors:** Shiyu Yan, Jinlang Lyu, Chunjie Yin, Wenhao Li, Yan Li, Hai-jun Wang

**Affiliations:** 1 Clinical Research Center for Children's Health and Diseases, Shandong Provincial Maternal and Child Health Care Hospital Affiliated to Qingdao University, Jinan, China; 2 Department of Maternal and Child Health, School of Public Health, Peking University, National Health Commission Key Laboratory of Reproductive Health, Peking University Health Science Center-Weifang Joint Research Center for Maternal and Child Health, Xueyuan road 38, Beijing, 312000, China, 86 13699145149; 3 Institute of Child Health Care, Jinan Children's Hospital, Jinan, China

**Keywords:** child, obesity, mobile health, intervention, adherence

## Abstract

**Background:**

Childhood obesity remains a major public health challenge. Mobile health (mHealth) interventions offer a scalable approach to support behavior change, but their effectiveness may depend on participant adherence. While both caregiver and child adherence are important, few studies have jointly examined caregiver app-use adherence and child behavioral adherence within digital obesity interventions.

**Objective:**

This study aimed to evaluate the independent and joint associations of caregiver app-use adherence and child behavioral adherence with obesity-related outcomes in a school-based mHealth intervention.

**Methods:**

We conducted a secondary analysis of 684 child-caregiver dyads in the intervention arm of the diet, exercise, and cardiovascular health—children cluster-randomized trial, implemented across 3 socioeconomically diverse regions in China. Caregiver app-use adherence was measured using app-recorded app-use frequency and total usage duration. Child behavioral adherence was assessed through weekly behavior monitoring scores covering diet, screen time, and physical activity. Linear mixed models estimated associations between adherence levels and changes in anthropometric and physical fitness outcomes over 9 months.

**Results:**

Higher caregiver app-use frequency and duration were both significantly associated with reductions in children’s obesity-related indicators, such as BMI (β=−0.19; *P*=.005), BMI *z*-score (β=−0.08; *P*=.006), and body fat percentage (BFP) (β=−0.70; *P*=.005) for frequency. Greater child behavioral adherence was associated with lower BFP (β=−0.63; *P*=.01) and better physical fitness, including shorter shuttle run time (β=−3.63; *P*<.001) and longer standing long jump distance (β=2.88; *P*=.005). App-use frequency and duration showed a significant interaction for rope-skipping performance (*P*=.05). In joint adherence analyses, children in the high caregiver-high child adherence group showed the most favorable changes, including lower BMI (β=−0.22; *P*=.02), lower BFP (β=−1.18 percentage points; *P*=.001), and 7.45 additional rope-skipping repetitions (*P*=.003), compared with the low caregiver-low child adherence group.

**Conclusions:**

In this secondary analysis of an mHealth app-assisted childhood obesity intervention, higher caregiver app-use adherence and child behavioral adherence were associated with more favorable changes in weight status and physical fitness. These findings underscore the importance of coengagement in digital health programs and highlight the potential of app-supported, family-engaged strategies for childhood obesity prevention.

## Introduction

Childhood overweight and obesity have become critical global public health challenges. According to the World Health Organization (WHO), the prevalence of overweight and obesity among children and adolescents aged 5‐19 years has risen sharply from 4% in 1975 to 20% in 2022 [1]. In China, the obesity rate among primary and middle school students increased from 0.1% in 1985 to 9.6% in 2019, a 75.6-fold increase [2]. Childhood obesity is associated with adverse physical and psychological health consequences during childhood and elevates the risk of hypertension, type 2 diabetes, and certain cancers in adulthood [3]. Effective prevention in early life is therefore essential to curbing the obesity epidemic and reducing long-term health impacts and economic burdens.

Although numerous intervention studies on childhood obesity have been conducted worldwide, their results have been inconsistent, even when similar strategies were used [4,5]. One key but often overlooked factor related to intervention outcomes is participant adherence, defined as the extent to which participants comply with the study protocol [6]. Poor adherence can reduce the delivered intervention dose and complicate the interpretation of intervention findings. However, adherence remains underreported in obesity intervention research: a systematic review found that only 37% of high-quality studies included adherence data [7]. In addition, when reported, adherence was mostly used to assess feasibility rather than to quantitatively examine its association with intervention outcomes [8]. This gap limits understanding of how adherence is related to intervention outcomes and hinders the identification of scalable, evidence-informed strategies.

The emergence of mobile health (mHealth) technologies offers new opportunities to deliver, monitor, and tailor obesity interventions in real time [9,10]. Smartphone apps enable continuous health education, behavior tracking, and personalized feedback, potentially enhancing engagement and sustainability. This digital approach also helps address a longstanding challenge in intervention research: the repeated and simultaneous measurement of adherence. In childhood obesity interventions, adherence involves both intervention implementers, such as caregivers or teachers, and intervention participants, namely children themselves [11]. However, traditional methods have struggled to capture this process; caregiver adherence is often understudied due to measurement challenges and incomplete data [12]. Likewise, child adherence is frequently reported as a behavioral outcome rather than being examined as a process indicator related to intervention outcomes. Consequently, evidence on how caregiver app-use adherence and child behavioral adherence are jointly associated with obesity-related outcomes remains scarce. The automated data capture capabilities of mHealth platforms now allow caregiver app use and child behavioral adherence to be measured repeatedly, facilitating analysis of their independent and joint associations with health outcomes.

To address these gaps, we leveraged a mobile app-supported, multicomponent childhood obesity intervention. The app automatically recorded caregiver app-use data, while children’s behavioral adherence was tracked weekly through structured monitoring. This secondary analysis of the intervention arm of the diet, exercise, and cardiovascular health—children (DECIDE-Children) cluster randomized trial aimed to evaluate how caregiver app-use adherence and child behavioral adherence, individually and jointly, were associated with obesity-related outcomes.

## Methods

### Study Design and Secondary Analysis Sample

This study was a secondary analysis of the intervention arm of the DECIDE-Children trial conducted in 3 socioeconomically diverse regions of China: Beijing, Changzhi (Shanxi Province), and Urumqi (Xinjiang Province). A total of 24 schools (8 schools per region) with Grade 4 students were randomly assigned (1:1) to the intervention or control group after baseline assessments. The 9-month multifaceted intervention (from September 2018 to June 2019) targeted both children and their environments by promoting a healthy diet and physical activity and by engaging schools and families to support children’s behavioral changes, with family involvement strengthened through the “Eat Wisely, Move Happily” smartphone app. Details of the trial protocol have been published [13]. The reporting of this study followed the CONSORT-EHEALTH (Consolidated Standards of Reporting Trials of Electronic and Mobile Health Applications and Online Telehealth) guidelines; the completed checklist is provided in Checklist 1.

The primary effectiveness analysis of the DECIDE-Children trial has been published previously [14]. Compared with the control group, the intervention group had a significantly greater reduction in BMI from baseline to the end of the trial, with a mean between-group difference in BMI change of −0.46 (95% CI −0.67 to −0.25; *P*<.001). The prevalence of obesity decreased by 27.0% relative to baseline in the intervention group, compared with 5.6% in the control group. The intervention also improved other adiposity outcomes, dietary, sedentary, and physical activity behaviors, and obesity-related knowledge, although no significant changes were observed in moderate-to-vigorous physical activity, physical fitness, or blood pressure. The present study focused on 684 child-caregiver dyads in the intervention arm to examine how caregiver app use and child behavioral adherence were associated with obesity-related outcomes within the intervention group.

### Ethical Considerations

The original trial was approved by the Peking University Institutional Review Board (IRB00001052-18021). Written informed consent was obtained from the parents or legal guardians of all participating children before enrollment. Participation was voluntary, and participants were free to withdraw from the study at any time. The original ethics approval covered secondary analyses of the collected data; therefore, no additional informed consent was required for the present secondary analysis. To protect participant privacy and confidentiality, the database used for analysis contained no directly identifiable information, and coded identifiers were used instead.

### Intervention

The intervention was designed based on a social-ecological framework and implemented at school, family, and individual levels. At the school level, the intervention included establishing school policies to support obesity prevention and providing health education lectures and training for teachers. Teachers then delivered health education sessions to students during regular school health education classes. The core messages focused on healthy eating and physical activity, including avoiding overeating and sugar-sweetened beverages, reducing high-energy foods and sedentary time, and increasing physical activity.

At the family level, caregivers received health education materials and were encouraged to support children’s behavioral changes at home, particularly by improving the home food environment and supporting physical activity outside of school. Family involvement was strengthened through a mobile app, which was installed on caregivers’ smartphones to avoid increasing children’s screen time. Through the app, caregivers could receive information on obesity prevention, record their children’s weekly dietary and physical activity behaviors, and view their children’s assessment and feedback information.

At the individual student level, the intervention included health education sessions, reinforcement of in-school physical activity, and regular monitoring of weight and height. Physical education teachers encouraged students to engage in moderate-to-vigorous physical activity during school, and trained project staff or trained teachers measured children’s weight and height regularly according to standardized procedures. These data were entered into the intervention system and used to generate feedback on children’s weight status and weight-management progress.

The mobile app supported the intervention through 4 main modules: information diffusion, behavior monitoring, weight management, and assessment and feedback. In the behavior-monitoring module, caregivers were asked to observe and/or inquire about their child’s weekly diet, screen time, and physical activity behaviors and report this information in the app, preferably together with the child. In the assessment and feedback module, the app automatically generated feedback based on children’s weight-monitoring data and behavior-monitoring records. Caregivers and students could access these assessment results and feedback through the app, which was intended to help families understand their children’s weight-management status and reinforce targeted behavior changes during the 9-month intervention.

### Adherence Measurement via Mobile App

#### Caregiver App-Use Adherence

The mobile app included 4 main modules: information diffusion, behavior monitoring, weight management, and assessment and feedback (Table S1 in Multimedia Appendix 1). Caregiver app use referred to app-recorded use of the “Eat Wisely, Move Happily” mobile app and was used to measure caregiver adherence to the app-based components of the intervention. When caregivers used any function of the app, the background system automatically recorded the student ID, the app-use event, and the usage duration. App-use frequency was defined as the total number of app-use events recorded across all modules, and app-use duration was defined as the cumulative time spent using the app. In the present analysis, these 2 app-recorded indicators were used to quantify caregiver app-use adherence.

#### Child Behavioral Adherence

Child behavioral adherence was defined as the extent to which children followed the targeted weekly diet, screen time, and physical activity recommendations. It was assessed through the behavior-monitoring module. According to the intervention protocol, caregivers were asked to complete the behavior-monitoring questionnaire once per week, for a total of 35 time points during the intervention. Caregivers were instructed to observe and/or inquire about their child’s dietary and physical activity behaviors during the preceding week. The questionnaire was pushed through the app every Sunday at 8:00 PM, when caregivers were generally more available and could review the child’s behaviors from the preceding week together with the child.

The behavior score included 7 components aligned with the intervention goals: drinking sugary beverages, eating Western fast food, eating fried food, eating unhealthy snacks, overeating, screen time of more than 1 hour, and at-home exercise. Overeating was explained to caregivers during the health education component as eating beyond satiety or eating noticeably more than the child’s usual amount. Scores were assigned according to the weekly frequency of each behavior (Table S2 in Multimedia Appendix 1). For each item, 5 points indicated meeting the recommended standard, and lower scores indicated poorer adherence to the recommended behavior. The maximum weekly score was 35 points, with higher scores indicating better behavioral adherence by the child. Missing behavior-monitoring data accounted for approximately 10% of data each month and were imputed using a 2-step approach, as described in a previously published analysis of the same intervention data [15].

### Outcome Measures

Obesity-related outcomes included adiposity indicators and physical fitness measures. Adiposity indicators included changes in BMI, BMI *z*-score, waist circumference (WC), and body fat percentage (BFP) from baseline to the 9-month follow-up. Height was measured using a stadiometer (Huateng GMCS-1; Beijing Xindong Huateng Sports Equipment Co, Ltd), and weight was measured using a lever scale (Wujin RGT-140). BMI was calculated as weight in kilograms divided by height in meters squared. The BMI *z*-score was calculated according to the WHO growth reference using each child’s age and sex [16]. WC was measured using a MyoTape, and BFP was assessed using a body composition analyzer (Tanita MC-780 MA). Physical fitness measures included the 50 m × 8 shuttle run, standing long jump, and 1-minute rope skipping, which were assessed by trained outcome assessors according to standardized procedures. All outcomes were measured at baseline and after the 9-month intervention.

### Statistical Analyses

#### Descriptive Analysis

Baseline characteristics of students and their caregivers were summarized. Continuous variables were presented as means with SDs for normally distributed data, whereas categorical variables were reported as frequencies and percentages.

#### Association Between Caregivers’ and Children’s Adherence and Obesity-Related Outcomes

Caregiver app-use adherence was measured using two app-recorded indicators: (1) app-use frequency, defined as the total number of app-use events recorded when caregivers used any function of the app, and (2) app-use duration, defined as the cumulative time spent using the app. Child behavioral adherence was calculated as the average weekly score for diet, screen time, and physical activity behaviors reported through the behavior-monitoring module. Each adherence indicator was classified in two ways: (1) binary classification using the median (low vs high); (2) quartile classification (Q1: ≥ upper quartile, Q2: median to < upper quartile, Q3: lower quartile to < median, Q4: < lower quartile).

Linear mixed-effects models were used to evaluate the associations between adherence levels and changes in obesity-related outcomes, including BMI, BMI *z*-score, WC, BFP, 50 m × 8 shuttle-run time, standing long-jump distance, and 1-minute rope-skipping performance. All models were adjusted for child’s age, gender, parental age, parental BMI, parental education level, and the corresponding baseline measure of each outcome. A random intercept for class was included to account for within-class clustering. For quartile-based analyses, trend tests were conducted by treating the quartile variable as an ordinal predictor.

To examine the relative importance of app-use frequency and app-use duration, participants were categorized into 4 subgroups based on median splits of both app-use frequency and app-use duration: high frequency and high duration, high frequency and low duration, low frequency and high duration, and low frequency and low duration. This 2×2 classification allowed us to compare different app-use patterns. A formal interaction term between app-use frequency and app-use duration was included in the model to assess whether their joint association with outcomes differed from their individual associations.

#### Joint Associations of Caregiver and Child Adherence and Trend Analyses

To investigate joint associations, three joint adherence variables were constructed by combining caregiver and child adherence indicators: (1) app-use duration × child behavioral adherence, (2) app-use frequency × child behavioral adherence, and (3) combined app-use pattern × child behavioral adherence. Each joint variable was categorized into 3 groups: both low adherence, mixed adherence, and both high adherences. The “both low adherence” group served as the reference group. Linear mixed-effects models were used to assess the associations between joint adherence groups and changes in obesity-related outcomes. Covariates and random effects were consistent with the previous models. Trend tests were conducted by treating the joint adherence groups as an ordinal variable. To assess potential variation in associations, interaction terms between caregiver and child adherence were included in the models.

### Sensitivity Analyses

To examine the robustness of the findings, sensitivity analyses were conducted by excluding students who were primarily cared for by grandparents, as caregiver type may be related to app-use patterns, behavior monitoring, and response to the intervention.

The results were considered statistically significant at a 2-sided *P*<.05. Statistical analyses were carried out using R software (version 4.1.2; creators: John Chambers and colleagues; location: Jersey City, NJ, USA).

## Results

### Baseline Characteristics

A total of 684 students and their caregivers were included in the analysis. Baseline characteristics are presented in Table 1. Among the students, 343 were boys and 341 were girls. In addition, 627/683 (91.8%) students were of Han ethnicity. Most students were only children (408/678, 60.2%). The mean (SD) BMI and BMI *z*-score were 18.54 (3.70) and 0.70 (1.44), respectively. At baseline, 158 (23.1%) students had obesity, and 266 (38.9%) students had overweight or obesity.

**Table 1.** The baseline characteristics of children and their caregivers.

Characteristics	Values
Students	
Gender, n (%)	
Boy	343 (50.1)
Girl	341 (49.9)
Nation, n (%)^a^	
Han	627 (91.8)
Other	56 (8.2)
Single child, n (%)^a^	
Yes	408 (60.2)
No	270 (39.8)
Age, mean (SD)	9.62 (0.35)
BMI, mean (SD)	18.54 (3.70)
BMI *z* score, mean (SD)	0.70 (1.44)
With obesity, n (%)	
Yes	158 (23.1)
No	526 (76.9)
With overweight or obesity, n (%)	
Yes	266 (38.9)
No	418 (61.1)
Waist circumference (cm), mean (SD)	65.15 (10.23)
Waist-to-height ratio, mean (SD)	0.86 (0.06)
BFP^c^, mean (SD)	20.71 (10.43)
Caregivers, mean (SD)	
Mother age (y)	37.87 (4.24)
Father age (y)	39.88 (4.74)
Father BMI	24.20 (4.30)
Mother BMI	21.91 (4.04)
Fathers with overweight or obesity, n (%)^a^	
Yes	364 (57.2)
No	272 (42.8)
Mothers with overweight or obesity, n (%)^a^	
Yes	162 (24.7)
No	495 (75.3)
Fathers with obesity, n (%)^a^	
Yes	70 (11.0)
No	566 (89.0)
Mothers with obesity, n (%)^a^	
Yes	38 (5.8)
No	619 (94.2)
Primary caregiver, n (%)	
Mother	487 (71.2)
Father	163 (23.8)
Nonparents	34 (5.0)
Father’s education level, n (%)^a^	
High^b^	362 (54.4)
Low^b^	303 (45.6)
Mother’s education level, n (%)^a^	
High^b^	397 (59.8)
Low^b^	267 (40.2)
Mother with the job, n (%)^a^	
Yes	555 (84.2)
No	104 (15.8)

a There is a missing value, so the synthesis is not 684, and the missing value appears to be missing at random.

b “High” means a college degree or above, and “low” means less than a college degree.

c BFP: body fat percentage.

Among caregivers, the mean ages of mothers and fathers were 37.87 (SD 4.24) and 39.88 (SD 4.74) years, respectively. The prevalences of obesity or overweight were 5.8% (n=38) and 24.7% (n=162) among mothers and 11.0% (n=70) and 57.2% (n=364) among fathers. Most primary caregivers were mothers (n=508, 74.3%), whose education levels were slightly higher than those of fathers (higher education: n=397, 59.8% vs n=362, 54.4%). Additionally, 84.2% (n=555) of mothers were employed.

### Association Between Caregiver App-Use Adherence and Obesity-Related Outcomes

Caregiver app-use adherence was quantified using 2 app-recorded measures: app-use frequency and app-use duration. For app-use frequency, the corresponding median values were Q1, 32 (IQR 2‐44) events; Q2, 57 (IQR 45‐66) events; Q3, 81 (IQR 67‐103) events; and Q4, 133 (IQR 104‐529) events. For app-use duration, the quartile ranges were Q1, 17.40 (IQR 1.04‐24.58) minutes; Q2, 31.10 (IQR 24.59‐37.64) minutes; Q3, 46.60 (IQR 37.65‐58.77) minutes; and Q4, 82.20 (IQR 58.78‐240.04) minutes.

In the dichotomized analysis (Table 2), higher app-use frequency was associated with more favorable changes in BMI (β=−0.19, 95% CI −0.32 to −0.06; *P*=.005), BMI *z*-score (β=−0.08, 95% CI −0.13 to −0.02; *P*=.006), BFP (β=−0.70, 95% CI −1.19 to −0.21; *P*=.005), and rope-skipping performance (β=5.66, 95% CI 2.01-9.30; *P*=.002). Higher app-use duration was associated with lower BMI (β=−0.15, 95% CI −0.28 to −0.01; *P*=.03), BMI *z*-score (β=−0.06, 95% CI −0.12 to −0.01; *P*=.02), BFP (β=−0.51, 95% CI −1.01 to −0.02; *P*=.04), and better rope skipping performance (β=4.99, 95% CI 1.32-8.65; *P*=.008). No significant associations were found for WC, shuttle run time, or standing long jump distance.

**Table 2.** Association between binary caregiver and child adherence measures and child health outcomes.

Outcome	App usage frequency		App usage duration		Behavior score	
	β (95% CI)	*P* value	β (95% CI)	*P* value	β (95% CI)	*P* value
△^a^BMI	−0.19 (−0.32 to −0.06)	.005	−0.15 (−0.28 to −0.01)	.03	−0.07 (−0.20 to 0.07)	.34
△BMI *z*-score	−0.08 (−0.13 to −0.02)	.006	−0.06 (−0.12 to −0.01)	.02	−0.02 (−0.07 to 0.04)	.57
△WC^b^	−0.34 (−0.84 to 0.16)	.19	−0.33 (−0.84 to 0.17)	.18	−0.36 (−0.86 to 0.15)	.17
△BFP^c^	−0.70 (−1.19 to −0.21)	.005	−0.51 (−1.01 to −0.02)	.04	−0.63 (−1.13 to −0.14)	.01
△shuttle run time	−0.66 (−2.72 to 1.40)	.53	−0.28 (−2.35 to 1.79)	.79	−3.63 −5.66 to −1.60)	<.001
△ standing long jump	−0.01 (−2.06 to 2.04)	≥.99	0.28 (−1.79 to 2.34)	.79	2.88 (0.85 to 4.91)	.005
△1-minute rope jumping	5.66 (2.01 to 9.30)	.002	4.99 (1.32 to 8.65)	.008	4.06 (0.45 to 7.67)	.03

a △ indicates the change from baseline to the end of the intervention, calculated as the postintervention value minus the baseline value.

b WC: waist circumference.

c BFP: body fat percentage.

Caregiver adherence measures refer to app-recorded app-use frequency and total usage duration. Child adherence refers to the average weekly caregiver-child behavior-monitoring score for diet, screen time, and at-home exercise. *P* values were estimated from linear mixed-effects models adjusted for age, gender, parental age, parental BMI, parental education level, and the baseline value of each outcome, with class as a random effect.

In the quartile-based analysis (Table S3 in Multimedia Appendix 1), a higher app-use frequency was significantly associated with more favorable changes in BMI, BMI *z*-score, WC, BFP, and rope-skipping performance (*P* for trend =.01, .01, .02, .01, and .009, respectively). Higher app-use duration was also significantly associated with more favorable changes in BMI, BMI *z*-score, WC, and rope-skipping performance (*P* for trend=.02, .01, .03, and .004, respectively), whereas the association with BFP was not statistically significant (*P* for trend =.08).

### Association Between Child Adherence and Obesity-Related Outcomes

For child behavioral adherence scores, quartile ranges were Q1, 29.0 (IQR 22.25‐29.95); Q2, 30.80 (IQR 29.96‐31.54); Q3, 32.40 (IQR 31.55‐33.14); and Q4, 34.10 (IQR 33.15‐35). In the dichotomized analysis (Table 2), higher child behavioral adherence was associated with lower BFP (β=−0.63, 95% CI −1.13 to −0.14; *P*=.01), shorter shuttle-run time (β=−3.63, 95% CI −5.66 to −1.60; *P*<.001), longer standing long-jump distance (β=2.88, 95% CI 0.85-4.91; *P*=.005), and greater rope-skipping counts (β=4.06, 95% CI 0.45-7.67; *P*=.03). No statistically significant associations were observed between child behavioral adherence and BMI, BMI *z*-score, or WC.

Further analyses using quartile-based child adherence groups also revealed a dose-response relationship for several outcomes (Table S4 in Multimedia Appendix 1). Compared with children in the lowest quartile, those in the highest quartile of behavioral adherence had more favorable changes in BFP (β=−0.78, 95% CI −1.49 to −0.08; *P* for trend =.009), shuttle run time (β=−4.17, 95% CI −7.05 to −1.28; *P* for trend =.008), standing long jump distance (β=4.07, 95% CI 1.20-6.93; *P* for trend =.004), and rope-skipping counts (β=6.14, 95% CI 1.04-11.25; *P* for trend =.010). No significant trends were observed for BMI, BMI *z*-score, or WC.

### Comparison of App-Use Patterns: Frequency Vs Duration

As shown in Table 3, children whose caregivers belonged to group 4 (high frequency and high duration) showed the most favorable changes in several obesity-related outcomes, including BMI (β=−0.21, 95% CI −0.36 to −0.06; *P*<.01), BMI *z*-score (β=−0.09, 95% CI −0.15 to −0.03; *P*<.05), and BFP (β=−0.76, 95% CI −1.32 to −0.21; *P*<.01), compared with the reference group (low frequency and low duration). Favorable changes in physical fitness, particularly rope skipping, were also observed across multiple groups with higher app-use frequency or duration.

**Table 3.** Joint associations of app-use frequency and duration with child health outcomes.

Outcome	Group 1^a^ (Ref)	Group 2^b^, β (95% CI)	Group 3^c^, β (95% CI)	Group 4^d^, β (95% CI)	*P* value for interaction
△BMI	1.00	−0.11 (−1.16 to 2.53)	0.00 (−0.23 to 0.23)	−0.21 (−0.36 to −0.06)^e^	.55
△BMI *z*-score	1.00	−0.03 (−0.13 to 0.06)	−0.00 (−0.10 to 0.09)	−0.09 (−0.15 to −0.03)^f^	.43
△WC^g^	1.00	.04 (−0.83 to 0.92)	0.06 (−0.82 to 0.95)	−0.42 (−0.99 to 0.15)	.40
△BFP^h^	1.00	−0.47 (−1.34 to 0.39)	−0.00 (−0.87 to 0.86)	−0.76 (−1.32 to −0.21)^e^	.64
△shuttle run time	1.00	−1.25 (−4.82 to 2.32)	−0.34 (−4.00 to 3.31)	−0.59 (−2.92 to 1.73)	.70
△standing long jump	1.00	1.55 (−2.03 to 5.12)	0.19 (−2.12 to 2.49)	2.25 (−1.34 to 5.84)	.16
△1-minute rope jumping	1.00	6.74 (2.64 to 10.83)^e^	7.06 (0.66 to 13.46)	8.52 (2.26 to 14.77)^e^	.048

a Group 1: low frequency and low duration.

b Group 2: high frequency and low duration (frequent but short sessions).

c Group 3: low frequency and high duration (infrequent but longer sessions).

d Group 4: high frequency and high duration.

e *P*<.01.

f *P*<.05.

g WC: waist circumference.

h BFP: body fat percentage.

To further compare app-use frequency and duration patterns, we compared group 2 (high frequency and low duration) with Group 3 (low frequency and high duration). No statistically significant differences were found across the health indicators. Although the interaction term between app-use frequency and duration was not significant for most outcomes, it was significant for rope-skipping performance (*P*=.048), suggesting a possible joint association of frequent and prolonged app use with this physical fitness outcome.

### Joint Associations of Caregiver and Child Adherence and Trend Analyses

To explore the combined association of caregiver app-use adherence and child behavioral adherence, we constructed three joint adherence variables: app-use duration × child behavioral adherence, app-use frequency × child behavioral adherence, and combined app-use pattern × child behavioral adherence. Each joint variable was categorized into three groups: both low adherence, mixed adherence, and both high adherences.

**Figure 1.**
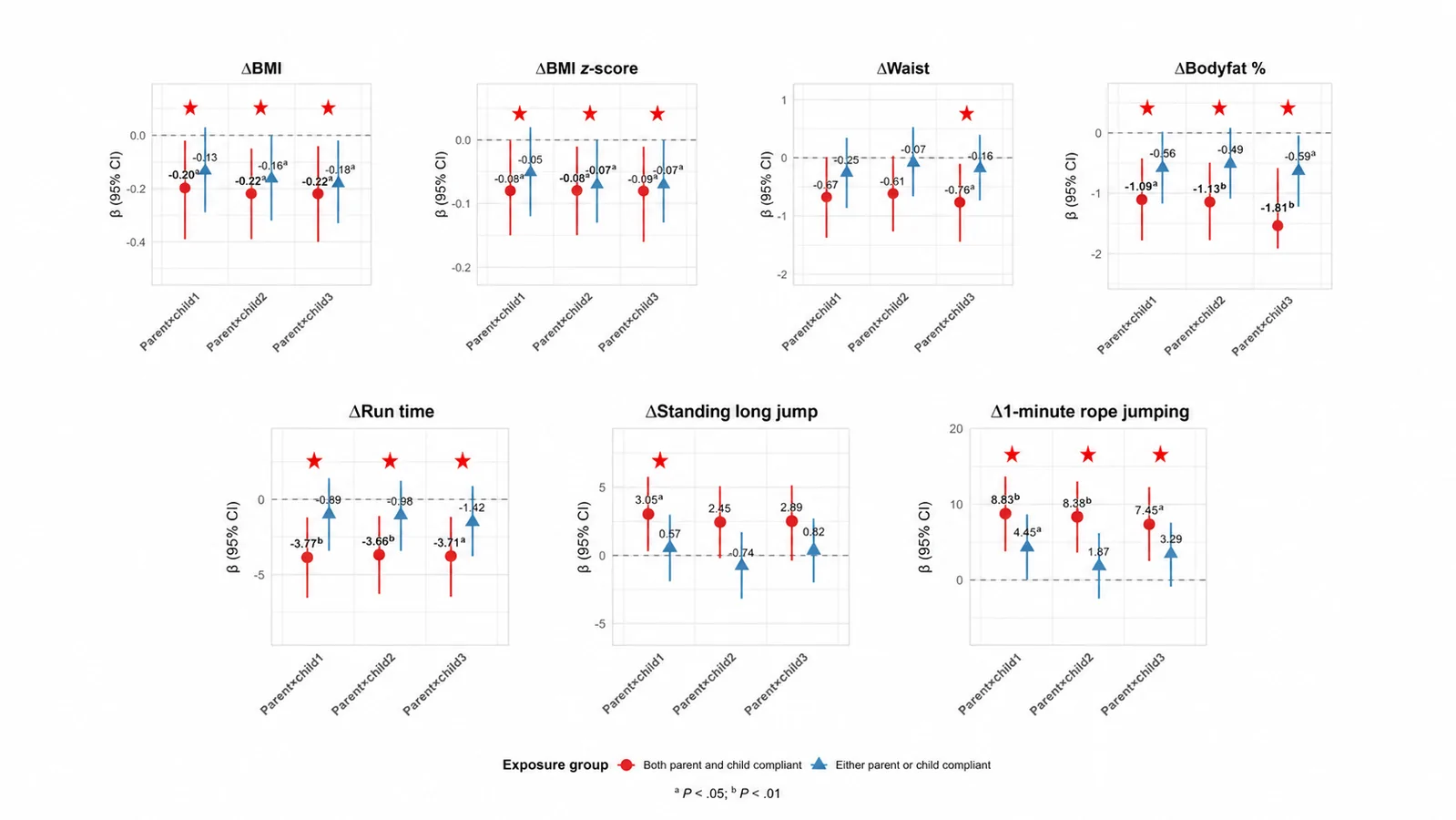
Joint associations of caregiver app use and child behavioral adherence with obesity-related outcomes.

As shown in Figure 1 and Table S5 in Multimedia Appendix 1, children in both high-adherence groups showed the most favorable outcomes across several obesity-related and physical-fitness indicators. Compared with the both low-adherence group, the both high-adherence group had more favorable changes in BMI (eg, app-use pattern × child adherence: β=−0.22, 95% CI −0.40 to −0.04; *P*=.02), BMI *z*-score (β=−0.08, 95% CI −0.16 to −0.01; *P*=.03), and BFP (β=−1.18, 95% CI −1.85 to −0.51; *P*=.001). Physical fitness measures also showed favorable changes; for example, the both high adherence group had a shorter shuttle run time (β=−3.71, 95% CI −6.50 to −0.93; *P*=.009) and more rope-skipping repetitions (β=7.45, 95% CI 2.50 to 12.40; *P*=.003) in the app-use pattern × child adherence model.

The figure shows adjusted β coefficients and 95% CIs for the joint associations of caregiver app use and child behavioral adherence with seven obesity-related outcomes. Three caregiver app-use indicators were examined: app-use duration, app-use frequency, and combined high-frequency or high-duration app use. For each indicator, participants were classified into 3 groups: both low adherence (reference), mixed adherence, and both high adherence. Blue triangles indicate mixed adherence, and red circles indicate high adherence. Red stars indicate significant trends across adherence groups (*P* for trend<.05).

Trend analyses demonstrated significant dose–response relationships for most outcomes, indicating that higher levels of joint caregiver–child adherence were associated with progressively greater improvements in both adiposity and physical fitness indicators. Significant interaction effects between caregiver and child adherence were observed for BMI and standing long jump performance, suggesting that the association of one adherence component with these outcomes may vary according to the level of the other adherence component.

### Sensitivity Analysis

To assess the robustness of the findings, we conducted sensitivity analyses by excluding participants who were primarily cared for by grandparents. The results, as shown in Table S6 in Multimedia Appendix 1, were largely consistent with the main analyses, supporting the stability of our conclusions.

## Discussion

### Principal Findings

To our knowledge, this was the first study to simultaneously examine caregiver app-use adherence, child behavioral adherence, and their joint associations with obesity-related outcomes in a multicomponent, app-supported childhood obesity intervention. Caregiver app-use adherence was associated mainly with adiposity-related outcomes: higher app-use frequency and longer app-use duration were consistently associated with more favorable changes in BMI, BMI *z*-score, and BFP, with graded patterns across adherence levels. Child behavioral adherence was more strongly associated with physical fitness indicators. However, given the large number of statistical comparisons performed, these statistically significant associations should be interpreted as exploratory and hypothesis-generating rather than confirmatory. This caution is particularly important for interaction analyses. The statistically significant interaction between app-use frequency and duration for rope-skipping performance suggests that both dimensions of app use may be relevant to this physical fitness outcome, but this finding requires confirmation in future studies. Similarly, the observed interaction terms between caregiver and child adherence suggest that their joint associations may differ from either component alone, although these findings should also be regarded as exploratory. Children with both high caregiver app-use adherence and high child behavioral adherence tended to show more favorable outcomes across several indicators, suggesting the potential importance of family coadherence in digital childhood obesity interventions.

### Comparison With Prior Work

Participant adherence has been widely recognized as a key factor related to the effectiveness of obesity interventions. Previous studies have adopted various metrics to assess adherence and have generally reported more favorable outcomes among more adherent participants. One study used caregivers’ questionnaire completion as a measure of adherence and found that children of caregivers who complied experienced greater weight loss [17]. Another study grouped participants according to the number of educational sessions attended and found that participants in the adherent group (attended ≥3 sessions) showed significantly greater improvements in obesity-related indicators compared with those with lower attendance [18]. In interventions using interactive voice response technology, higher engagement, measured by the number of completed calls, was associated with reductions in child weight, BMI, and BMI *z*-scores [19,20]. Our findings provide additional evidence that caregiver app-use adherence and child behavioral adherence were both associated with more favorable obesity-related outcomes. These results support the importance of considering engagement and adherence in the design and implementation of childhood obesity interventions.

Both app-use frequency and app-use duration appear to be important components of digital intervention dose. Our findings suggest that these two dimensions were jointly associated with obesity-related outcomes. Although childhood obesity interventions often require substantial cumulative contact time, such as the suggested 26 hours in the first intervention year [21], the challenge lies in delivering this dose in formats acceptable to families. Evidence indicates that brief but frequent interactions may be more feasible and potentially more effective than longer, infrequent sessions. A dietary education study replaced traditional intensive dietary counseling with short, frequent telephone contacts, an approach that elicited positive participant feedback and meaningful lifestyle improvements [22]. Given increasing time constraints in modern family life, adherence becomes a central barrier. Transitioning from long-format counseling to high-frequency, short-duration digital touchpoints may improve adherence while preserving intervention dose, offering a scalable and family-centered strategy for real-world childhood obesity interventions.

The sensitivity analysis excluding children primarily cared for by grandparents showed generally similar associations, suggesting that the main findings were unlikely to be driven solely by this relatively small caregiver subgroup. One possible explanation is that grandparents caring for primary school-aged children may still have sufficient familiarity with smartphones to participate in app-based intervention activities. Nevertheless, caregiver heterogeneity should be considered in future mHealth interventions. Parents and grandparents may differ in smartphone familiarity, app-use habits, and digital health literacy, which could influence intervention engagement and behavior monitoring. Prior reviews have shown that older adults’ use of eHealth or health technologies can be affected by self-efficacy, knowledge, support, technology usability, and socioeconomic factors [23,24]. Future mHealth interventions may therefore need tailored support for caregivers with different levels of technological familiarity.

The joint high-adherence pattern in both caregivers and children was associated with the most favorable changes in obesity-related and physical fitness outcomes, highlighting the importance of engaging both parties in digital health interventions. The different patterns observed for caregiver and child adherence are consistent with the design of our multicomponent intervention. The intervention app was primarily designed for caregivers to receive nutritional guidance. This may explain why caregiver app-use adherence was more strongly associated with weight-related indicators, such as BMI and BFP. By providing actionable dietary advice and tools for home environment modification, the app supported caregivers—who often influence food purchasing, meal preparation, and children’s eating opportunities—in implementing healthier eating practices for their children. This interpretation is consistent with evidence that parent or family involvement and parent-targeted dietary support are important components of childhood obesity interventions [25,26]. In contrast, children’s behavioral adherence was more strongly associated with physical fitness improvements. This aligns with the student-level component of our intervention, which integrated enhanced, structured physical activity into the school curriculum. Higher child behavioral adherence may reflect more consistent practice of recommended dietary, screen time, and physical activity behaviors [27]. Overall, our results suggest that childhood obesity interventions may benefit from supporting both caregiver involvement and children’s adherence to targeted health behaviors. Future interventions should incorporate strategies that help caregivers create supportive home environments while encouraging children to practice and sustain healthy dietary and physical activity behaviors.

### Strengths and Limitations

The main strength of this study lies in the richness and objectivity of its process evaluation data and the objective recording of caregiver app use. Caregiver app-use data were automatically recorded by the mobile app, providing objective, time-stamped information on app-use frequency and duration. Children’s health behaviors were repeatedly and systematically collected through the caregiver-child behavior-monitoring module, allowing us to examine children’s behavioral adherence over the intervention period. Compared with studies relying solely on qualitative process evaluation or retrospective end-of-study self-reports, the repeated app-based data collection in this study provided more detailed quantitative information for adherence analysis [28]. Moreover, we considered adherence from both the perspectives of the intervention implementers (caregivers) and the intervention subjects (children). By simultaneously evaluating caregiver app-use adherence and children’s behavioral adherence, we were able to examine the interplay between the 2 and better understand how selected adherence-related processes were associated with intervention outcomes.

Several limitations should be acknowledged. First, the baseline prevalence of obesity and overweight or obesity in our analytical sample was relatively high compared with national estimates for Chinese children of similar age. This should be interpreted cautiously because the present analysis was based on a trial-based regional sample rather than a nationally representative sample. Previous Chinese surveillance studies have reported substantial geographic, urban-rural, sex, and socioeconomic variation in childhood overweight and obesity, with some school populations showing prevalence estimates higher than national averages [29]. Second, although intervention fidelity encompasses multiple components—including adherence, dose, quality of delivery, participant responsiveness, and program differentiation [30], the present analysis focused on the dose of caregiver app-use and child behavioral adherence. Process evaluation data from the DECIDE-Children trial showed that several school-level fidelity components, such as delivery of health education sessions and implementation of school-based activities, consistently achieved high levels (>80%) with limited variability across schools and participants [14]. Therefore, these indicators were less suitable for explaining individual differences in obesity-related outcomes in the present analysis. Nevertheless, other potentially informative fidelity or engagement indicators were not fully captured, such as teacher implementation quality, children’s direct participation in school-based activities, and children’s engagement with intervention content. Thus, our findings should be interpreted as reflecting selected adherence-related processes rather than the full implementation fidelity of the intervention. Third, the temporal ordering between caregiver app engagement, child behavioral adherence, and child obesity-related outcomes could not be firmly established in this secondary analysis. Although adherence indicators were assessed during the intervention period and outcomes were evaluated over follow-up, caregiver engagement may both influence and be influenced by children’s behavioral changes or weight-related progress. For example, caregivers of children who showed more favorable changes may have been more motivated to continue using the app, whereas caregivers facing greater difficulties may have disengaged over time. Therefore, reverse causality cannot be excluded, and the observed associations should not be interpreted as causal effects of adherence. Fourth, child behavioral adherence was measured using caregiver-child behavior monitoring reported through the app. Although caregivers were instructed to observe and/or inquire about their child’s weekly behaviors and preferably complete the report together with the child, this measure remains proxy-reported and may reflect caregiver perceptions. In addition, caregiver app use and caregiver-reported child behavioral adherence were derived from the same respondent, which may introduce common-method bias and inflate the observed associations between caregiver and child adherence indicators. Caregivers who used the app more frequently may also have been more diligent in reporting children’s behaviors, which could introduce reporting-related correlation. Future studies should incorporate more direct and multidimensional measures of child engagement, including school-level participation records, direct activity monitoring, and children’s own reports where appropriate. Finally, because multiple statistical comparisons were conducted across several adherence indicators and obesity-related outcomes, individual statistically significant findings, particularly interaction terms, should be interpreted as exploratory and hypothesis-generating rather than confirmatory.

### Implications and Future Research

Future studies should evaluate adherence to better interpret the true effectiveness of complex interventions. In our study, children with higher caregiver and child adherence levels showed more favorable changes, suggesting that intervention effects may be underestimated when adherence is not considered—an issue aligned with the concept of “type III error,” in which poor implementation rather than ineffective intervention leads to null findings [31]. Evidence from prior childhood obesity interventions similarly shows that meaningful benefits emerge when implementation fidelity is moderate to high [32]. Therefore, routine collection of detailed adherence data and integration of implementation evaluation frameworks are essential for accurately assessing intervention impact and informing policy and practice [33].

### Conclusion

In this secondary analysis of an mHealth app-assisted childhood obesity intervention, higher caregiver app-use adherence and children’s behavioral adherence were associated with more favorable changes in weight status and physical fitness. These findings underscore the importance of coengagement in digital health programs and highlight the potential of app-supported, family-engaged strategies for childhood obesity prevention.

## Supplementary material

10.2196/89548 Multimedia Appendix 1 App intervention, adherence measures, and associations of caregiver and child adherence with child health outcomes.

10.2196/89548 Checklist 1 CONSORT-EHEALTH checklist.

## Abbreviations

BFP: body fat percentage
CONSORT-EHEALTH: Consolidated Standards of Reporting Trials of Electronic and Mobile Health Applications and Online Telehealth
DECIDE-Children: diet, exercise, and cardiovascular health—children
mHealth: mobile health
WC: waist circumference
WHO: World Health Organization
