# Supplementary material for: Caregiver App Use and Child Behavioral Adherence in Relation to Obesity-Related Outcomes in an mHealth App-Assisted Childhood Obesity Intervention: Secondary Analysis of a Cluster Randomized Trial
Source: JMIR Mhealth Uhealth. 2026 Sep 24;14:e89548. doi: 10.2196/89548 (PMC13608226; doi:10.2196/89548)
Supplement: Supplementary file 2 [file mhealth-v14-e89548-s001.docx]

**Supplementary materials**

**Table S1. Four app modules, detailed content, and completion frequency**.

| **Modules** | **Contents** | **Frequency** |
| --- | --- | --- |
| Information diffusion | Obesity-prevention information was regularly sent to caregivers and students. | At least 15 times throughout the intervention period |
| Behavior monitoring | Caregivers recorded the children's dietary and physical activity behaviors and completed the behavior questionnaire weekly. | 35 times |
| Health education homework | Caregivers and children completed health education homework outside school hours and uploaded records of completion to the mobile app. | At least 15 times throughout the intervention period |
| Assessment and feedback | Based on children’s weight and behavior-monitoring information, the mobile app automatically generated comprehensive monthly assessments and feedback, including weight-monitoring results, weight-change assessments, and dietary and physical activity behavior assessments. Caregivers and children could access the results through the mobile app. | At least 7 times throughout the intervention period |

**Table S2. Scores corresponding to the weekly frequency of dietary and physical activity behaviors**

| **Part** | **5 points** | **4 points** | **3 points** | **2 points** | **1 point** |
| --- | --- | --- | --- | --- | --- |
| Drinking sugary beverages | 0 | 1-2 times | 3-4 times | 5-6 times | More than 7 times |
| Eating Western-style fast food | 0 | 1-2 times | 3-4 times | 5-6 times | More than 7 times |
| Eating fried food | 0 | 1-2 times | 3-4 times | 5-6 times | More than 7 times |
| Eating unhealthy snacks | 0 | 1-2 times | 3-4 times | 5-6 times | More than 7 times |
| Overeating | 0 | 1-2 times | 3-4 times | 5-6 times | More than 7 times |
| Screen time of more than 1 hour | 0 | 1-2 days | 3-4 days | 5-6 days | More than 7 days |
| At-home exercise | Following the protocol | >30 min/day of other forms of exercise | - | <30 min/day of other forms of exercise | No exercise |

**Table S3. Associations between quartiles of caregiver adherence and child health outcomes**

|  |  |  | App usage frequency | | |  | | App usage duration | |
| --- | --- | --- | --- | --- | --- | --- | --- | --- | --- |
| Outcome |  |  | n | β (95% CI) | P for trend |  | n | β(95% CI) | P for trend |
| △BMI | Group1 |  | 171 | Reference | .01 |  | 172 | Reference | .02 |
|  | Group2 |  | 171 | 0.05 (-0.13, 0.24) |  |  | 170 | -0.05 (-0.23, 0.13) |  |
|  | Group3 |  | 171 | -0.02 (-0.21, 0.16) |  |  | 173 | -0.14 (-0.33, 0.05) |  |
|  | Group4 |  | 171 | -0.30 (-0.49, -0.12)** |  |  | 169 | -0.21 (-0.40, -0.02)* |  |
| △BMI Z-score | Group1 |  | 171 | Reference | .01 |  | 172 | Reference | .01 |
|  | Group2 |  | 171 | 0.01 (-0.07, 0.08) |  |  | 170 | -0.03 (-0.10, 0.05) |  |
|  | Group3 |  | 171 | -0.02 (-0.10, 0.06) |  |  | 173 | -0.07 (-0.14, 0.01) |  |
|  | Group4 |  | 171 | -0.13 (-0.20, -0.05)* |  |  | 169 | -0.09 (-0.17, -0.01)* |  |
| △WC | Group1 |  | 171 | Reference | .02 |  | 172 | Reference | .03 |
|  | Group2 |  | 171 | -0.38 (-1.08, 0.32) |  |  | 170 | -0.37 (-1.07, 0.31) |  |
|  | Group3 |  | 171 | -0.09 (-0.80, 0.61) |  |  | 173 | -0.15 (-0.87, 0.56) |  |
|  | Group4 |  | 171 | -1.02 (-1.73, -0.31)* |  |  | 169 | -0.94 (-1.66, -0.22)* |  |
| △BFP | Group1 |  | 171 | Reference | .01 |  | 172 | Reference | .08 |
|  | Group2 |  | 171 | -0.11 (-0.59, 0.80) |  |  | 170 | -0.06 (-0.75, 0.62) |  |
|  | Group3 |  | 171 | -0.53 (-1.23, 0.17) |  |  | 173 | -0.59 (-1.30, 0.11) |  |
|  | Group4 |  | 171 | -0.77 (-1.48, -0.07)* |  |  | 169 | -0.50 (-1.22, 0.20) |  |
| △time for shuttle run | Group1 |  | 171 | Reference | .09 |  | 172 | Reference | .56 |
|  | Group2 |  | 171 | -2.15 (-5.04, 0.75) |  |  | 170 | -1.32 (-4.18, 1.55) |  |
|  | Group3 |  | 171 | -0.45 (-3.37, 2.48) |  |  | 173 | -0.90 (-3.83, 2.05) |  |
|  | Group4 |  | 171 | -3.27 (-6.20, -0.34)* |  |  | 169 | -1.10 (-4.06, 1.87) |  |
| △standing long jump | Group1 |  | 171 | Reference | .29 |  | 172 | Reference | .38 |
|  | Group2 |  | 171 | 2.89 (0.00, 5.76) |  |  | 170 | 1.87 (-0.98, 4.71) |  |
|  | Group3 |  | 171 | 0.64 (-2.27, 3.54) |  |  | 173 | 0.81 (-2.12, 3.72) |  |
|  | Group4 |  | 171 | 2.47 (-0.45, 5.38) |  |  | 169 | 1.79 (-1.14, 4.73) |  |
| △one-minute rope jumping | Group1 |  | 171 | Reference | .009 |  | 172 | Reference | .004 |
|  | Group2 |  | 171 | 1.70 (-3.48, 6.87) |  |  | 170 | 2.64 (-2.41, 7.70) |  |
|  | Group3 |  | 171 | 6.34 (1.12, 11.55)* |  |  | 173 | 5.35 (0.20, 10.52)* |  |
|  | Group4 |  | 171 | 6.83 (1.61, 12.03)* |  |  | 169 | 7.55 (2.29, 12.80)* |  |

Abbreviations: BFP, body fat percentage; BMI, body mass index; WC, waist circumference.

*P<.05; **P<.01.

Adherence (app usage frequency or duration) increased progressively from Group 1 to Group 4. Groups were defined by quartiles: Group 1, < lower quartile; Group 2, lower quartile to < median; Group 3, median to < upper quartile; and Group 4, ≥ upper quartile. Group 1 served as the reference group.

**Table S4. Associations between quartiles of child adherence and child health outcomes**

| **Outcome** | **Group** | **n** | **β（95% CI）** | **P for trend** |
| --- | --- | --- | --- | --- |
| **△BMI** | Group1 | 171 | Reference | .64 |
|  | Group2 | 170 | -0.03 (-1.06, 2.66) |  |
|  | Group3 | 170 | -0.15 (-0.34, 0.04) |  |
|  | Group4 | 171 | -0.01 (-0.20, 0.18) |  |
| **△BMI Z score** | Group1 | 171 | Reference | .77 |
|  | Group2 | 170 | -0.01 (-0.09, 0.06) |  |
|  | Group3 | 170 | -0.04 (-0.12, 0.04) |  |
|  | Group4 | 171 | 0.00 (-0.08, 0.07) |  |
| **△WC** | Group1 | 171 | Reference | .18 |
|  | Group2 | 170 | -0.29 (-0.99, 0.40) |  |
|  | Group3 | 170 | -0.60 (-1.31, 0.11) |  |
|  | Group4 | 171 | -0.42 (-1.14, 0.29) |  |
| **△BFP** | Group1 | 171 | Reference | .009 |
|  | Group2 | 170 | -0.26 (-0.95, 0.43) |  |
|  | Group3 | 170 | -0.76 (-1.46, -0.05)* |  |
|  | Group4 | 171 | -0.78 (-1.49, -0.08)* |  |
| **△time for shuttle run** | Group1 | 171 | Reference | .008 |
|  | Group2 | 170 | -1.31 (-4.14, 1.52) |  |
|  | Group3 | 170 | -4.44 (-7.28, -1.60)** |  |
|  | Group4 | 171 | -4.17 (-7.05, -1.28)** |  |
| **△standing long jump** | Group1 | 171 | Reference | .004 |
|  | Group2 | 170 | 1.10 (-1.73, 3.94) |  |
|  | Group3 | 170 | 2.84 (-0.06, 5.73) |  |
|  | Group4 | 171 | 4.07 (1.20, 6.93)** |  |
| **△one-minute rope jumping** | Group1 | 171 | Reference | .01 |
|  | Group2 | 170 | 3.44 (-1.65, 8.52) |  |
|  | Group3 | 170 | 5.55 (0.42, 10.68)* |  |
|  | Group4 | 171 | 6.14 (1.04, 11.25)* |  |

Abbreviations: BFP, body fat percentage; BMI, body mass index; WC, waist circumference.

*P<.05; **P<.01.

Child adherence (average weekly score of dietary and physical activity behaviors) increased progressively from Group 1 to Group 4. Groups were defined by quartiles: Group 1, < lower quartile; Group 2, lower quartile to < median; Group 3, median to < upper quartile; and Group 4, ≥ upper quartile. Group 1 served as the reference group.

**Table S5. Joint associations of caregiver and child adherence with child health outcomes and trend analyses**

| Outcome | Adherence | Beta(95% CI)# | P# | Beta(95% CI)* | P* | P for trend | P_interaction |
| --- | --- | --- | --- | --- | --- | --- | --- |
| BMI | Duration × Child | -0.13 (-0.29, 0.03) | .12 | -0.20 (-0.39, -0.02) | .03 | .03 | .69 |
| BMI | Number × Child | -0.16 (-0.32, -0.00) | .04 | -0.22 (-0.39, -0.05) | .01 | .01 | .04 |
| BMI | Usage × Child | -0.18 (-0.33, -0.02) | .02 | -0.22 (-0.40, -0.04) | .02 | .01 | .04 |
| BMI Z-score | Duration × Child | -0.05 (-0.12, 0.02) | .14 | -0.08 (-0.15, 0.00) | .046 | .046 | .70 |
| BMI Z-score | Number × Child | -0.07 (-0.13, -0.00) | .045 | -0.08 (-0.15, -0.01) | .03 | .03 | .33 |
| BMI Z-score | Usage × Child | -0.07 (-0.13, -0.00) | .04 | -0.08 (-0.16, -0.01) | .03 | .02 | .24 |
| Waist | Duration × Child | -0.25 (-0.86, 0.35) | .41 | -0.67 (-1.37, 0.02) | .06 | .06 | .74 |
| Waist | Number × Child | -0.07 (-0.66, 0.53) | .82 | -0.61 (-1.26, 0.05) | .07 | .07 | .35 |
| Waist | Usage × Child | -0.16 (-0.73, 0.41) | .59 | -0.76 (-1.44, -0.08) | .03 | .04 | .40 |
| BFP | Duration × Child | -0.56 (-1.16, 0.03) | .07 | -1.09 (-1.77, -0.41) | .002 | .002 | .94 |
| BFP | Number × Child | -0.49 (-1.08, 0.09) | .10 | -1.13 (-1.77, -0.49) | .001 | .001 | .77 |
| BFP | Usage × Child | -0.59 (-1.15, -0.03) | .04 | -1.18 (-1.85, -0.51) | .001 | .001 | .97 |
| Run Time | Duration × Child | -0.89 (-3.37, 1.60) | .48 | -3.77 (-6.61, -0.93) | .009 | .008 | .33 |
| Run Time | Number × Child | -0.98 (-3.43, 1.46) | .43 | -3.66 (-6.33, -0.99) | .007 | .007 | .41 |
| Run Time | Usage × Child | -1.42 (-3.76, 0.92) | .23 | -3.71 (-6.50, -0.93) | .009 | .01 | .43 |
| Jump | Duration × Child | 0.57 (-1.91, 3.06) | .65 | 3.05 (0.23, 5.87) | .03 | .03 | .37 |
| Jump | Number × Child | -0.74 (-3.17, 1.70) | .55 | 2.45 (-0.21, 5.10) | .07 | .07 | .047 |
| Jump | Usage × Child | 0.32 (-2.02, 2.66) | .79 | 2.39 (-0.39, 5.17) | .09 | .11 | .23 |
| Rope Skipping | App × Child | 4.45 (0.04, 8.86) | .048 | 8.83 (3.81, 13.85) | .001 | .001 | .98 |
| Rope Skipping | Number × Child | 1.87 (-2.45, 6.19) | .40 | 8.38 (3.64, 13.11) | .001 | .001 | .20 |
| Rope Skipping | Usage × Child | 3.29 (-0.86, 7.45) | .12 | 7.45 (2.50, 12.40) | .003 | .003 | .81 |

Adherence was categorized as both low (low caregiver adherence + low child adherence; reference), mixed (either caregiver or child adherence was high and the other was low), or both high (both caregiver and child adherence were high).

Duration refers to caregiver app usage duration; Number refers to caregiver app usage frequency; Usage refers to the combined pattern of high duration and high frequency.

β (95% CI) and P values were estimated using linear mixed-effects models adjusted for age, gender, parental age, parental BMI, parental education level, and baseline outcome value, with class included as a random effect.

P for trend was tested by treating the joint adherence groups as an ordinal variable (both low=1, mixed=2, both high=3). P for interaction is the P value for the multiplicative interaction term between caregiver and child adherence.

Abbreviations: BFP, body fat percentage; BMI, body mass index; WC, waist circumference.

**Table S6. Associations between binary caregiver and child adherence measures and child health outcomes, excluding participants primarily cared for by grandparents**

|  | APP time |  | APP usage times |  | Behavior score |  |
| --- | --- | --- | --- | --- | --- | --- |
| Outcome | Beta(95% CI) | P | Beta(95% CI) | P | Beta(95% CI) | P |
| △BMI | -0.12 (-0.26, -0.02) | .04 | -0.21 (-0.37, -0.05) | .009 | -0.06 (-0.22, 0.10) | .43 |
| △BMI Z score | -0.06 (-0.12, -0.01) | .02 | -0.08 (-0.14, -0.01) | .02 | -0.01 (-0.07, 0.06) | .85 |
| △WC | -0.14 (-0.69, 0.41) | .62 | -0.19 (-0.74, 0.35) | .48 | -0.42 (-0.96, 0.13) | .13 |
| △BFP | -0.49 (-1.10, 0.12) | .12 | -0.77 (-1.37, -0.16) | .01 | -0.78 (-1.39, -0.18) | .01 |
| △time for shuttle run | -0.34 (-2.58, 1.90) | .77 | -0.03 (-2.26, 2.20) | .98 | -4.46 (-6.62, -2.29) | <.001 |
| △standing long jump | -0.76 (-3.05, 1.52) | .51 | -0.75 (-3.03, 1.53) | .52 | 2.31 (0.07, 4.56) | .04 |
| △one-minute rope jumping | 3.60 (0.22, 6.98) | .04 | 5.38 (1.17, 9.60) | .01 | 4.17 (0.02, 8.33) | .049 |

Abbreviations: BFP, body fat percentage; BMI, body mass index; WC, waist circumference.
